# Interfacial Polarization Switching in Al_0.92_Sc_0.08_N/GaN Heterostructures Grown by Sputter Epitaxy

**DOI:** 10.1002/advs.202503827

**Published:** 2025-06-06

**Authors:** Niklas Wolff, Georg Schönweger, Md. Redwanul Islam, Ziming Ding, Christian Kübel, Simon Fichtner, Lorenz Kienle

**Affiliations:** ^1^ Department of Material Science Kiel University Kaiserstr. 2 D‐24143 Kiel Germany; ^2^ Kiel Nano, Surface and Interface Science (KiNSIS) Kiel University Christian‐Albrechts‐Platz 4 D‐24118 Kiel Germany; ^3^ Fraunhofer Institute for Silicon Technology (ISIT) Fraunhoferstr. 1 D‐25524 Itzehoe Germany; ^4^ Advanced Electron Microscopy in Materials Research, Institute of Nanotechnology (INT) Karlsruhe Institute of Technology (KIT) Hermann‐von‐Helmholtz‐Platz 1 D‐76344 Eggenstein‐Leopoldshafen Germany; ^5^ Karlsruhe Nano Micro Facility (KNMFi) Karlsruhe Institute of Technology (KIT) D‐76344 Eggenstein‐Leopoldshafen Germany

**Keywords:** AlScN, ferroelectrics, materials science, scanning transmission electron microscopy, wurtzite‐type ferroelectrics

## Abstract

The integration of ferroelectric nitride Al_1‐x_Sc_x_N onto GaN templates can enable enhanced functionality in novel high‐power transistors and memory devices. This requires a detailed understanding of ferroelectric domain structures and their impact on the electrical properties. In this contribution, the sputter epitaxy of highly coherent Al_0.92_Sc_0.08_N thin films grown on GaN approaching lattice‐matching conditions is demonstrated. Scanning transmission electron microscopy (STEM) investigations reveal polar domains and the mechanism of domain propagation upon ferroelectric switching. Atomic resolution imaging suggests that polarization inversion commences by an interfacial switching process in which the monolayer next to the interface already changes its polarization from the as‐grown M‐ to N‐polarity. The atomic configurations of this planar polarization discontinuity are identified and systematic changes of the electronic structure are revealed by electron energy loss spectroscopy (EELS). Moreover, persistent domains with M‐polarity are identified at the top Pt electrode interface after switching. These insights on the location and the atomic structure of ferroelectric domains in sputter deposited Al_0.92_Sc_0.08_N/GaN heterostructures are compared to metal organic chemical vapor deposition (MOCVD)‐grown films and discussed with respect to their defect structure. This knowledge will support the development of future non‐volatile memory devices and novel transistor structures based on ferroelectric nitride thin films via interface and defect engineering.

## Graphical abstract

Understanding the structural and electronic interface characteristics of ferroelectric Al_1‐x_Sc_x_N/GaN heterostructures is crucial for the development of novel transistor structures with integrated ferroelectric functionality. In this article, Niklas Wolff and co‐workers report on the interfacial switching mechanisms and reveal atomic and electronic reconstructions after ferroelectric switching of Al_0.92_Sc_0.08_N/GaN heterojunctions using atomic resolution scanning transmission electron microscopy (STEM) investigations.

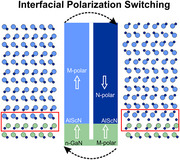

## 1 Introduction

The electronic properties resulting from the direction and magnitude of spontaneous polarization within multilayer structures of non‐centrosymmetric crystal structures are widely exploited in the field of epitaxial group III‐N heterojunctions. These heterojunctions are designed for optoelectronic applications or power electronic devices because of their exceptionally high electron or hole gas confinement and high mobilities created at these interfaces.^[^ ^1^, ^2^, ^3^ ^]^

The formation of a two‐dimensional electron gas (2DEG) between two semiconductors was initially achieved through the junction of GaAs and AlGaAs,^[^ ^4^, ^5^ ^]^ pioneering the development of contemporary AlGaN/GaN‐based high electron mobility transistor structures (HEMTs) for high‐power and high‐frequency applications.^[^ ^6^ ^]^ The design of HEMTs necessitates the optimization of interface properties as crystalline defects, including dislocations and lattice strain, can be detrimental to the electrical interface properties, e.g., the 2DEG.^[^ ^7^, ^8^, ^9^, ^10^, ^11^, ^12^ ^]^

In the recent past, significant advancements in the field of thin film ferroelectrics have led to the discovery of novel materials, including doped hafnium oxide,^[^ ^13^ ^]^ Sc‐ and B‐substituted AlN,^[^ ^14^, ^15^ ^]^ and Sc‐substituted GaN.^[^ ^16^, ^17^ ^]^ The integration of ferroelectric functionality into semiconductor heterostructures is intriguing for the manipulation of interfacial sheet charges.^[^ ^18^ ^]^ This can be achieved by dynamically modulating the direction of spontaneous polarization in a ferroelectric layer positioned adjacent to an AlGaN/GaN heterostructure^[^ ^19^, ^20^, ^21^, ^22^, ^23^ ^]^ or within the ferroelectric/GaN heterostructure itself.^[^ ^24^, ^25^ ^]^

The new wurtzite‐type ferroelectric nitrides, such as Al_1‐x_Sc_x_N, hold particular promise for facilitating the development of all‐nitride‐based ferroelectric (FE)‐HEMTs, featuring non‐volatile memory functionality and/or normally‐off operation. Theoretical calculations have demonstrated that polarization inversion can enhance electron sheet densities along Al_1‐x_Sc_x_N/GaN interfaces by more than one order of magnitude, particularly in the presence of sharp domain walls with head‐to‐head (H‐H) configuration at the interface.^[^ ^26^ ^]^ However, the existence and exploitation of such domain walls in these systems remain to be elucidated. Furthermore, the optimization of growth conditions and material parameters is imperative to achieve defect‐poor, high crystal quality and electrically tunable interfaces. Numerous studies have focused on lattice‐matching the Al_1‐x_Sc_x_N layer and the GaN‐based template. However, the lattice‐matched Sc composition of Al_1‐x_Sc_x_N on GaN has been reported to range from 9% to 18%.^[^ ^27^, ^28^, ^29^, ^30^, ^31^, ^32^, ^33^ ^]^ This presents a substantial challenge, given that identifying the appropriate lattice‐matched composition and its sensitivity to growth methods and conditions is imperative for the successful fabrication of high‐quality, strain‐free GaN‐based heterostructures incorporating ultra‐wide bandgap (ferroelectric) materials.

In conjunction with the advancement of enhanced growth conditions, it is necessary to gain a profound comprehension of the atomic configuration within the integrated ferroelectric nitride layer, particularly concerning crystalline or chemical imperfections and residual lattice strain. Moreover, a comprehensive understanding of ferroelectric polarization discontinuities in III‐N heterostructures is essential for the progression from fundamental research to device development. In this regard, the identification of the polarity at the unit cell level, the geometry of the polar domains, their propagation mechanisms, and the resulting local atomic structures at the domain walls before and after ferroelectric switching are pivotal parameters to be determined. This is particularly true for the polarization discontinuity at the interface, which is defining the electronic properties of the heterojunction.

With this contribution, we report on direct polarization investigations of ferroelectric nearly lattice‐matched Al_0.92_Sc_0.08_N/GaN heterostructures grown by physical vapor deposition (PVD ‐ sputter epitaxy) featuring high structural coherency. High‐resolution scanning transmission electron microscopy (HRSTEM) was performed to investigate the electric field‐induced domain structures after ex situ polarization inversion. We identify the stabilization of small M‐polar nucleation centers at the Pt/Al_0.92_Sc_0.08_N interface subsequent to the conversion of the spontaneous polarization to N‐polarity. Concurrently, a planar inversion domain boundary is formed at the GaN interface, marking the initial phase of the polarization inversion process. A comparison is made between these results and the domain structures and switching mechanisms observed in Al_0.86_Sc_0.14_N layers that have been grown by the metal organic chemical vapor deposition (MOCVD) method.^[^ ^34^ ^]^ The direct observation of domain boundary structures in sputter deposited Al_0.92_Sc_0.08_N thin films will have a significant impact on the research and development of novel devices in the field of non‐volatile memories and power electronics based on the ferroelectric switching properties of Al_1‐x_Sc_x_N thin films.

## 2 Results and Discussion

### 2.1 Nanostructure of Epitaxial Al_1‐x_Sc_x_N/GaN Heterostructures

**Figure 1:**
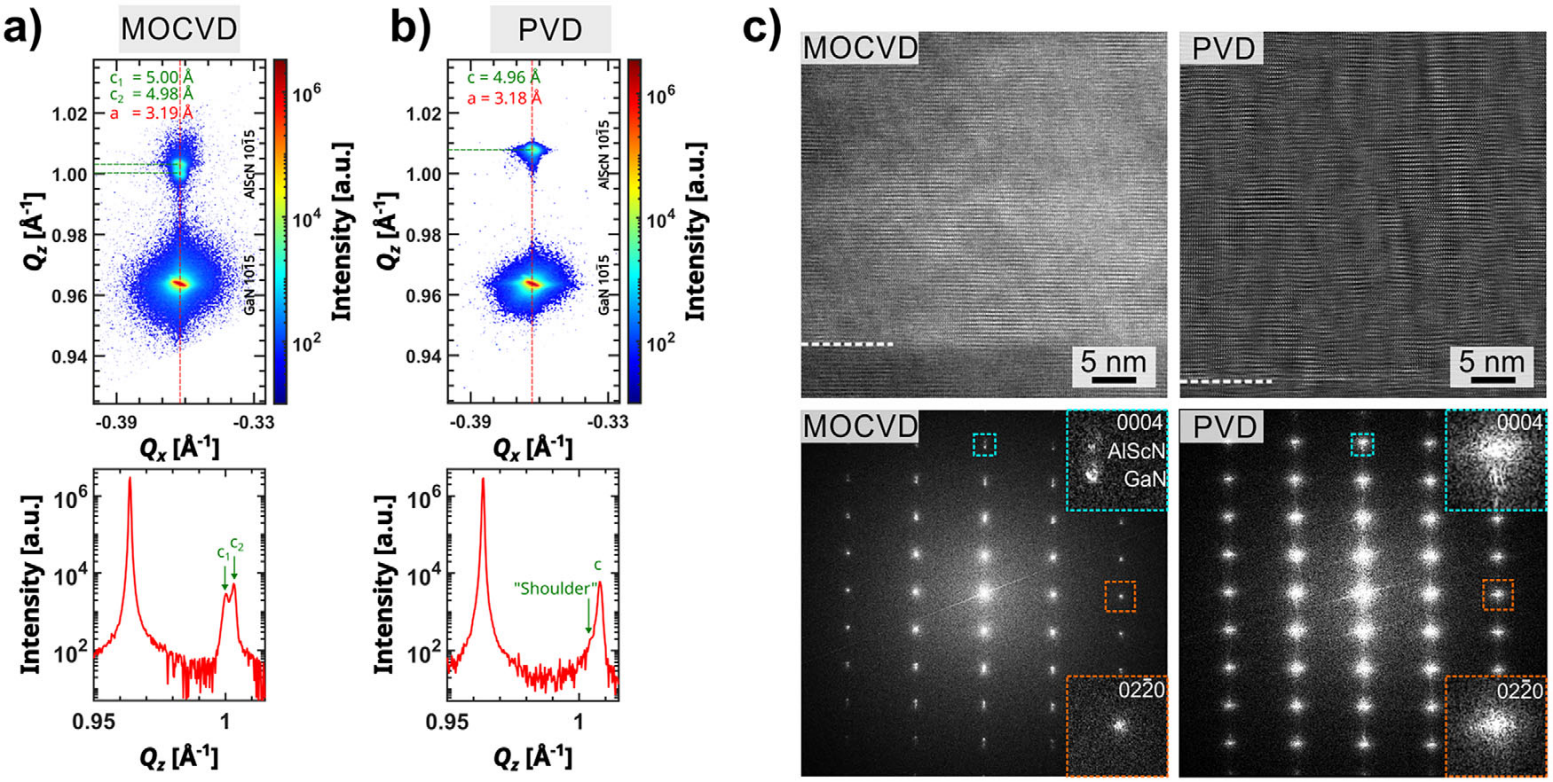
Comparison of nanostructure properties of ferroelectric and pseudomorphic Al_1‐x_Sc_x_N/GaN thin film heterostructures produced by MOCVD (Al_0.86_Sc_0.14_N , 230 nm) and PVD (Al_0.92_Sc_0.08_N , 110 nm). High‐resolution RSM (*Q*_*x*_ − *Q*_*z*_) of the AlScN and GaN 10$\bar{1}$5 reflections for a) MOCVD and b) PVD grown heterostructures. The intensity profiles show cutlines along *Q*_*z*_ at the center position *Q*_*x*_ of the reflections. c) HRTEM/FFT investigation of the crystalline properties at the GaN interface.

The crystal quality and epitaxy in the pseudomorphic (i.e., growing with same in‐plane lattice parameter) Al_1‐x_Sc_x_N/GaN heterostrucutures grown by MOCVD and sputter epitaxy is compared using high‐resolution reciprocal space maps (RSM) of the asymmetric Al_0.92_Sc_0.08_N and GaN 10$\bar{1}$5 reflections. These reflections have out‐of‐plane and in‐plane components which make them ideal candidates to determine the *c* and *a* lattice parameters of the wurtzite‐type structure. The latter gives access to compositional variation to achieve in‐plane lattice matched conditions. The average composition of the MOCVD film was previously determined to be Al_0.86_Sc_0.14_N by secondary ion mass spectroscopy and energy‐dispersive X‐ray spectroscopy (STEM‐EDS).^[^ ^33^ ^]^ By the evaluation of a series of sputter deposited films within the compositional range *x* ≈ 0.04–0.18, we found that films with a composition of *x* ≈ 0.08 and ≈ 0.11 determined by SEM‐EDS show the best structural quality with comparable narrow ω(0002) rocking curve full‐width at half maximum of ≈ 258 and ≈ 261 arcsec, compared to 252 arcsec for the MOCVD grown layer. This is in agreement with the Sc compositions reported for lattice matched conditions in literature.^[^ ^32^ ^]^ The Sc content of a thin cross‐section TEM lamella was determined to *x* ≈ 0.07 using STEM‐EDS which matches with *x* ≈ 0.08 determined using SEM‐EDS within the anticipated error margin of ≈ 2 at.%. The RSMs for the MOCVD Al_0.86_Sc_0.14_N and PVD Al_0.92_Sc_0.08_N grown heterostructures are displayed in **Figure** **1**a, b and show coherent alignment of the reflections on the *Q*_*x*_‐axis, indicating in‐plane lattice matching for both systems. However, a small deviation in the *a* lattice parameter for Al_0.92_Sc_0.08_N with 3.18 $\overset{˚}{A}$ from 3.19 $\overset{˚}{A}$ (GaN) is determined. The lower Sc content in the PVD film also gives rise to a smaller *c* lattice parameter of 4.96 $\overset{˚}{A}$ compared to the MOCVD grown layer with higher Sc content. A small asymmetry of the Al_0.92_Sc_0.08_N 10$\bar{1}$5 reflection intensity is observed in the *Q*_*z*_‐intensity profile. The shoulder at smaller *Q*_*z*_ values indicates the presence of areas with small tensile strain along the *c*‐axis, possibly at the GaN interface. Furthermore, a split of the 10$\bar{1}$5 reflection is observed for the MOCVD grown layer corresponding to *c*_1_ = 5.00 $\overset{˚}{A}$ and *c*_2_ = 4.98 $\overset{˚}{A}$. This observation might indicate a small compositional heterogeneity within the probed sample volume, which was not evidenced in our former TEM studies on a locally extracted lamella with finite dimensions of 10 µm *x* < 100 nm. Hence it might be possible that compositional domains exist on larger length scales.^[^ ^33^ ^]^

The crystal quality of the Al_1‐x_Sc_x_N/GaN interface for the as‐grown heterostructures is investigated by high‐resolution TEM (HRTEM). The Fourier‐filtered HRTEM micrographs recorded at an instrumental magnification of 300kX and their Fast‐Fourier‐Transforms (FFT) are presented in Figure 1c. Although the larger‐scale structural analysis via XRD show minimal differences in crystal quality between both thin films, the nanostructure analysis at the epitaxial interfaces show superior structural quality of the MOCVD‐grown layer. This is apparent from the FFT pattern that shows a very clear separation and a defined circular shape of the 0004 and 02$\bar{2}$0 intensities of the Al_0.86_Sc_0.14_N layer and the GaN template. In contrast, the FFT intensities of the sputtered Al_0.92_Sc_0.08_N layer are more diffuse and elliptically. The diffuse broadening of the reflections is related to higher structural disorder apparent from the phase contrast HRTEM micrograph. Nonetheless, the growth of a strongly *c*‐axis textured Al_0.92_Sc_0.08_N thin film with high structural coherence is achieved using sputter epitaxy at close to lattice‐matched film compositions.

**Figure 2:**
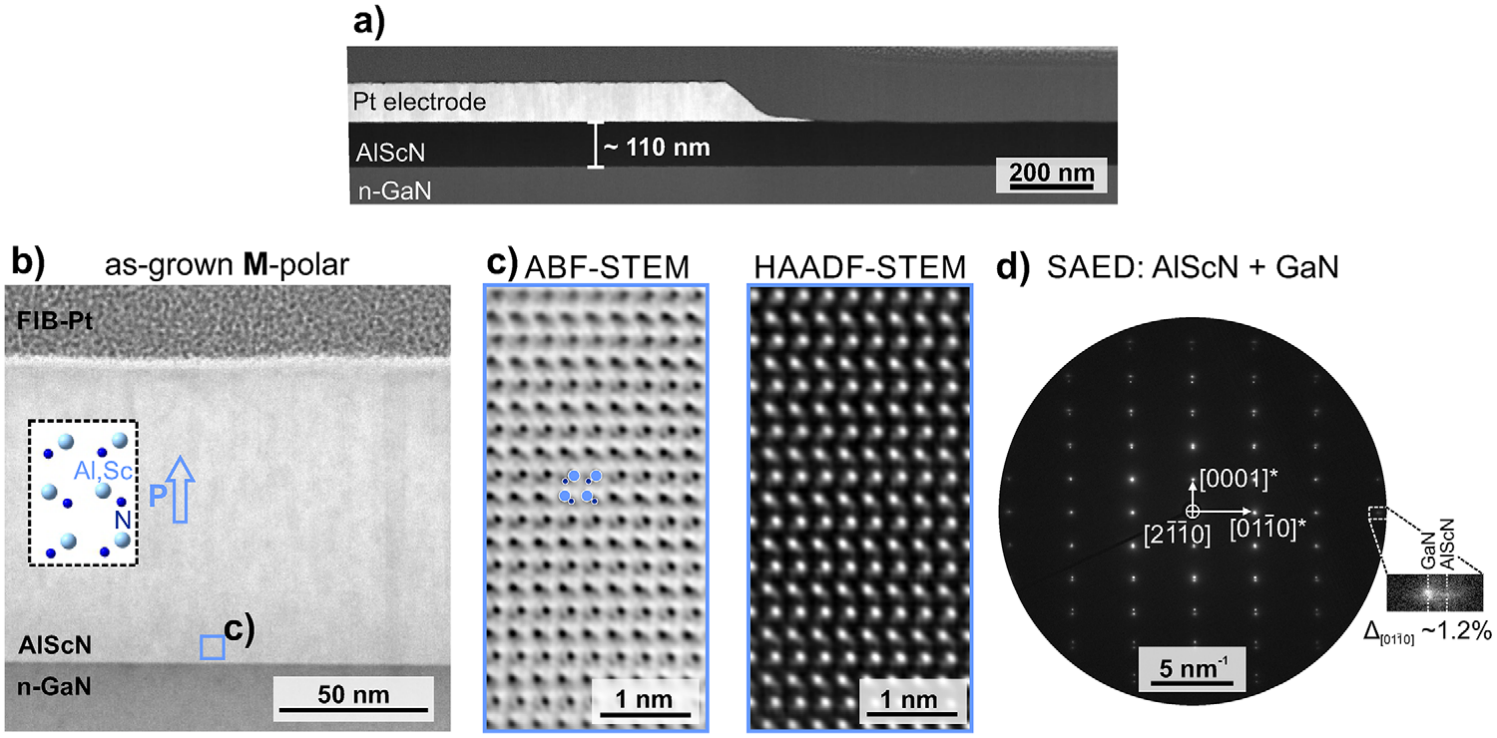
TEM investigation of the as‐grown thin film heterostructure. a) HAADF‐STEM image of the Pt/Al_0.92_Sc_0.08_N/n‐GaN capacitor in cross‐section. b) ABF‐STEM image of the as‐grown M‐polar Al_0.92_Sc_0.08_N/GaN region. The sketch shows the M‐polar atomic structure for the [2$\bar{1}$ $\bar{1}$0] crystal orientation. c) Atomic‐resolution HRSTEM images show M‐polar orientation near the GaN interface. d) SAED pattern in [2$\bar{1}$ $\bar{1}$0] zone axis orientation.

Next, we focus on the nanostructure and atomic scale investigations of the sputtered Al_0.92_Sc_0.08_N layer to determine the as‐grown film polarity using STEM. A cross‐section high‐angle annular dark‐field (HAADF)‐STEM image of the Pt/Al_0.92_Sc_0.08_N/n‐GaN capacitor structure is displayed in **Figure** **2**a showing a layer thickness of 110 nm and a smooth surface after structuring of the Pt electrode by ion‐beam etching. Figure 2b shows the annular bright‐field (ABF)‐STEM micrograph of the as‐grown part of the cross‐section lamella. On the atomic scale, the film appears highly coherent, which enabled the clear determination of metal (M = Al,Sc) and nitrogen atomic column positions from both ABF and HAADF‐STEM signals. Hence, M‐polarity is determined from the high‐resolution (HRSTEM) micrographs recorded on the as‐grown film showing an area close to the GaN interface in Figure 2c. The identified unipolar structure is different compared to layers with higher Sc content, where a switch to the N‐polar structure was observed at a distance of 40–50 nm from the interface in the as‐grown state.^[^ ^35^ ^]^ Analogous to the XRD examinations, a selected area electron diffraction (SAED) experiment was performed on the Al_0.92_Sc_0.08_N/GaN heterostructure. The investigated region was limited by the diffraction aperture having a virtual circular diameter of 250 nm. Figure 2d shows the single crystalline reflection patterns of the GaN and Al_0.92_Sc_0.08_N film in [2$\bar{1}$ $\bar{1}$0] zone axis. The observed splitting of the out‐of‐plane reflections along [0001]* agrees with the different *c*‐axis lattice parameters of GaN and Al_0.92_Sc_0.08_N. However, the separation of the 01$\bar{1}$0 in‐plane reflections of the GaN and Al_0.92_Sc_0.08_N layers show an averaged in‐plane lattice difference of about −1.2%. This difference is explained by strain relaxation and suggests non‐perfect lattice matched epitaxial growth conditions.

### 2.2 Ferroelectric Domains in Switched N‐Polar Al_0.92_Sc_0.08_N Thin Films

**Figure 3:**
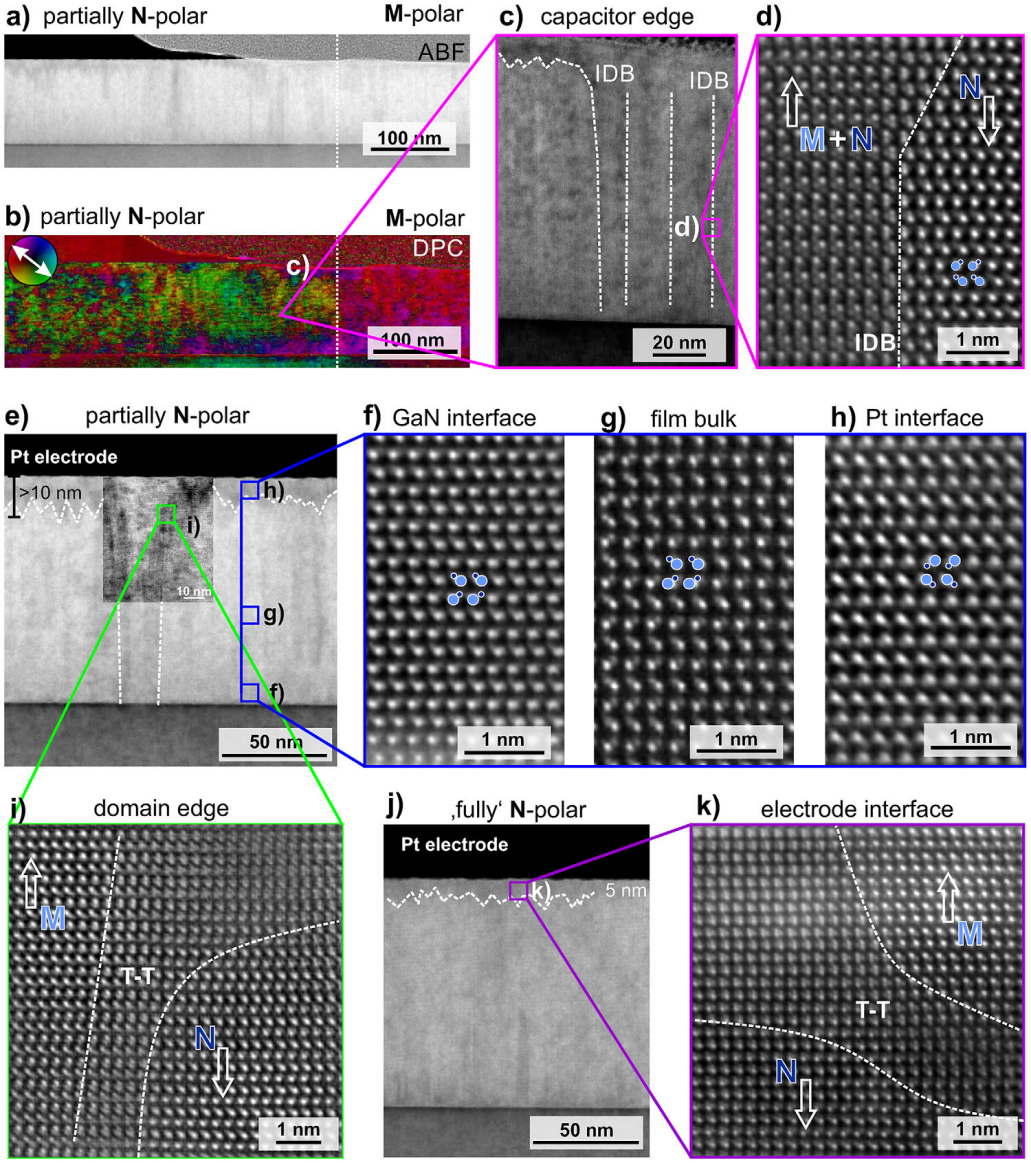
Investigation of ferroelectric domain structures after partial *E*_*switch*_ < *E*_*c*_ and complete *E*_*switch*_ > *E*_*c*_ polarization inversion. a) ABF‐STEM cross‐section image (inset: atomic structure projection with metal (Al, Sc) atoms in blue and nitrogen atoms in yellow. The polarization vector points toward the metal terminated unit cell. and b) DPC image of a sub‐saturation field biased Pt/Al_0.92_Sc_0.08_N/n‐GaN capacitor with the partially switched N‐polar layer (green: left) and the as‐grown M‐polar film (red: right). c) ABF‐STEM image showing the edge‐region of the switched capacitor with multiple inversion domain boundaries. d) HRSTEM image showing a vertical inversion domain boundaries at the position where a DPC contrast change was observed. e) ABF‐STEM micrograph showing the polar domain structure after partial switching. The dashed lines indicate domain boundaries. The inset image shows the boundaries of a vertical M‐polar domain at increased contrast. f–h) Investigation of local polarity by ADF‐HRSTEM at the GaN interface, the film bulk and the Pt interface. i) HRSTEM image of a curved T‐T domain wall at the upper edge of the remaining vertical M‐polar domain. j) ABF‐STEM image of a fully switched N‐polar layer. No vertical M‐polar domains are evident, but specific contrast pointing at pinned domains at the Pt electrode interface is observed. k) HRSTEM micrograph showing nanoscale residual M‐polar domains stabilized at the Pt‐interface after complete switching.

The ferroelectric domain structure in nearly lattice‐matched wurtzite‐type Al_1‐x_Sc_x_N thin films was investigated on cross‐section samples prepared on the edge of a switched capacitor structure, hence, allowing a direct comparison between as‐grown M‐polar and switched N‐polar regions within one sample.^[^ ^36^ ^]^ The presence of inversion domains can be observed from a combination of scattering contrast change in ABF‐STEM and atomic resolution imaging by HRSTEM.^[^ ^33^, ^34^ ^]^ Here, we compare as‐grown regions with regions to where non‐saturating and saturating voltage signals were applied. The applied electric fields were sufficient to invert the polarization direction partially and completely, respectively. By comparing the nanostructure of these film states the morphology, location and nucleation of ferroelectric domains can be observed, which provides access to the nanoscale switching mechanisms. The ferroelectric pretreatment is presented in Figure S1 (Supporting Information). In **Figure** **3** we provide an in‐depth analysis of partially‐switched and fully‐switched films after the application of a positive voltage signal to switch from the prior set M‐polar to the N‐polar state after cycling through 20 complete hysteresis loops. The ABF‐STEM micrograph presented in Figure 3a shows the partially switched layer within the capacitor structure in comparison to the as‐grown layer on the right. The ABF‐STEM micrograph contains contrast changes arising from local alterations of diffraction conditions. For example by ‐ minute tilts of crystal orientation or enhanced scattering as result of changed atomic density present at the overlap of M‐polar and N‐polar regions, as well as the presence of domain walls themselves. The differential phase contrast (DPC)‐STEM image (Figure 3b) maps the deflection of the focused electron beam by changes of the local electric field distribution, but can be corroborated by the change of diffraction conditions at defect structures.^[^ ^37^ ^]^ A distinct color change of about 180° from purple/red to green is observed at the edge of the capacitor indicating the inversion of polar orientation after switching. STEM and HRSTEM investigations (Figure 3c,d) of this edge region show the presence of multiple vertical domain boundaries and in‐between regions with superimposed polar structures.

A magnified overview of the partially switched film and its domain structure is presented in the ABF‐STEM image of Figure 3e. A clear change of contrast is observed in an area extending about 10 nm from the Pt electrode interface into the bulk of the film. The boundary of this layer appears fringed which is comparable to interfacial inversion domains observed before.^[^ ^34^ ^]^ The local polarity after partial switching is examined by HRSTEM revealing an overall N‐polar structure close to the GaN interface and the film bulk (cf. Figure 3f,g demonstrating ferroelectric polarization inversion). Further, residual M‐polar areas are observed at the Pt electrode interface (cf. Figure 3h). Moreover, the partially switched layer also exhibits vertically extending M‐polar regions, which have not yet been switched and are up to 15 nm wide. These domains extend over the whole Al_0.92_Sc_0.08_N film thickness, connecting the GaN interface with the residual M‐polar layer at the top interface. The vertical boundaries which separate the M‐polar and N‐polar regions are characterized by curved tail‐to‐tail (T‐T) domain walls as shown in Figure 3e,i.

In comparison to fully switched layers (Figure 3j), those vertical M‐polar domains bridging from interface to interface are not observed anymore and the pinned M‐polar layer is reduced in vertical dimension to about 5 nm small non‐connected M‐polar nuclei. In‐between the nuclei, areas showing the N‐polar motif and areas showing a superposition motif of both polarities are observed (Figure 3k).^[^ ^33^, ^38^ ^]^ In contrast to Al_0.86_Sc_0.14_N films grown by the MOCVD method, here we observe that M‐to‐N polarization inversion is initiated at the Al_0.92_Sc_0.08_N/GaN interface. This results in a sharp basal N‐ to M‐polar inversion domain boundary at the Al_0.92_Sc_0.08_N/GaN interface.^[^ ^33^, ^34^ ^]^ Another consequence of this is that sputtered Al_0.92_Sc_0.08_N films feature locally tail‐to‐tail (T‐T) domain walls, while MOCVD‐grown Al_0.86_Sc_0.14_N films only feature H‐H domain walls. Furthermore, the majority of the polar volume is already switched when applying sub‐saturating electric fields. However, a thin layer of non‐connected M‐polar regions remains stabilized at the Pt electrode even after applying apparently saturating electric fields (cf. Figure S1, Supporting Informarion).^[^ ^39^ ^]^

### 2.3 Interfacial Switching at The Al_0.92_Sc_0.08_N/GaN Interface

**Figure 4:**
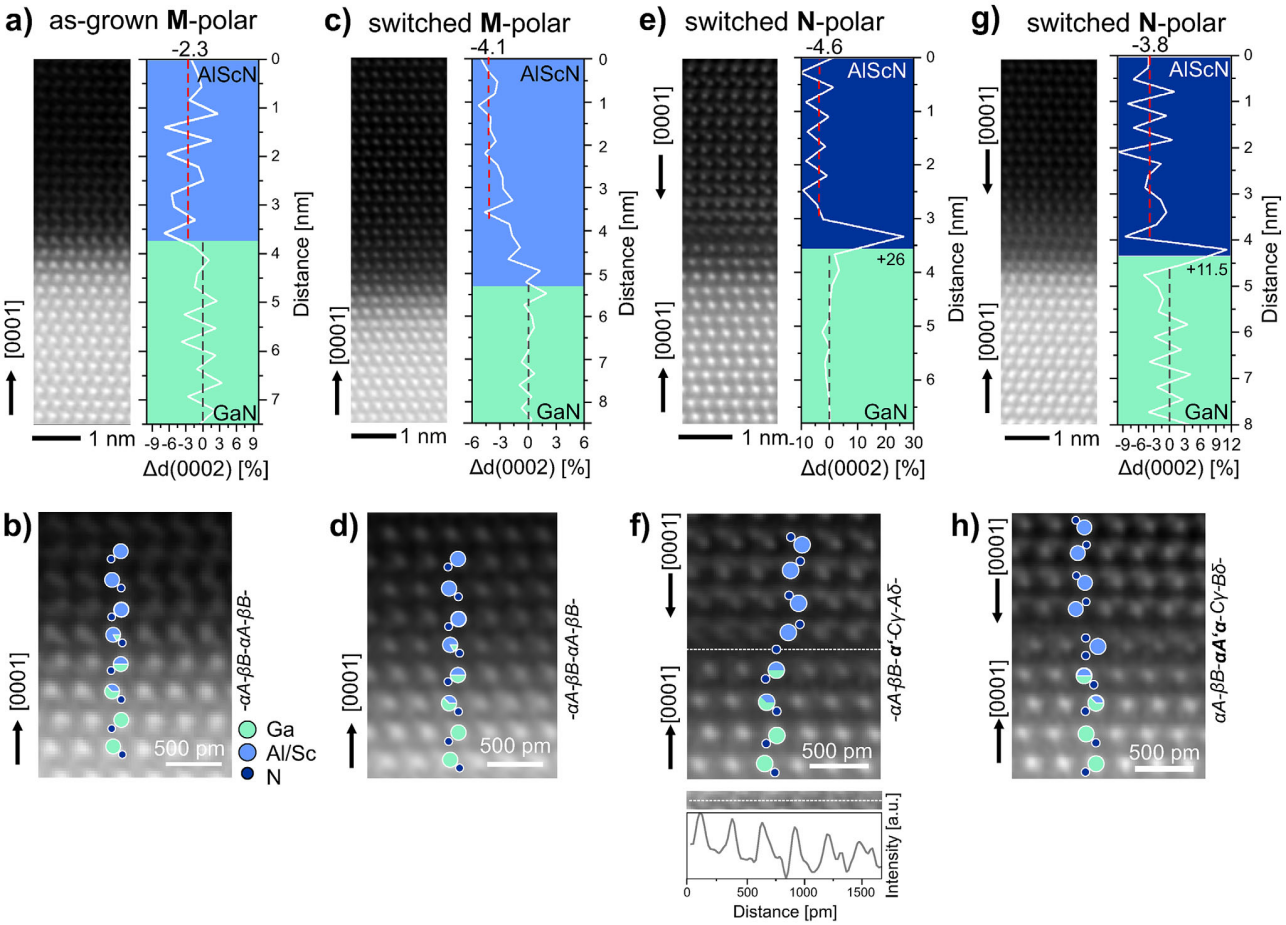
HRSTEM analysis of the atomic interface structures in as‐grown and switched Al_0.92_Sc_0.08_N/n‐GaN heterostructures. The STEM micrographs show the discovered atomic structures and profiles show the calculated relative out‐of‐plane strain Δ*d*(0002) across the basal‐plane monolayers. a,b) As‐grown M‐polar interface and c,d) switched M‐polar interface identified at the vertically extending M‐polar domain after partial switching. Hexagonal cation stacking of type *A‐B*_*n*_ is determined across the interface. (e,f) and (g,h) show the discovered interface configurations with interfacial H‐H polarization discontinuity after switching to the N‐polar state. The cation stacking sequence shows a discontinuity with cubic‐type (*A‐B‐C*) stacking and largely increased monolayer separation across the interface. The intensity profile in f) suggests the presence of an anion‐layer in the gap. The overall strain‐state of the Al_0.92_Sc_0.08_N layer is changed at all switched interface types, showing a relaxation from ‐2.3% to about ‐4% difference with respect to GaN.

From the HRSTEM micrograph shown in Figure 3f, the formation of a horizontal inversion domain boundary is suggested by the observation of complete polarization inversion close to the GaN interface. Therefore, the atomic structures at the Al_0.92_Sc_0.08_N/GaN interface prior and after ferroelectric switching experiments were examined. HRSTEM micrographs of the interface prior to ferroelectric switching are presented in **Figure** **4**a and as magnified view in 4b showing the highly coherent atomic structure across the interface. The gradual change of the atomic mass specific Z‐contrast in the HAADF image suggests intermixing of Al/Sc and Ga atomic positions across a few monolayers. Using the peak finding method from the Atomap Python library,^[^ ^40^ ^]^ the atomic positions of the metal cations (Ga and Al,Sc) were determined from the HAADF‐STEM images and assigned to individual (0002)‐monolayers. The relative separation between the Al_0.92_Sc_0.08_N monolayers Δ*d*(0002) at the interface was calculating relative to the average spacing between the GaN monolayers.

In Figure 4a the HRSTEM micrograph of the as‐grown interface and the relative out‐of‐plane strain profile are presented. The difference in calculated average monolayer spacing between the Al_0.92_Sc_0.08_N monolayers is Δ*d*(0001) = −2.3%, which belongs to a locally larger *c*‐lattice parameter than expected in comparison to the −4.4% difference calculated for the film bulk using the RSM. This difference in out‐of‐plane strain at the interface and the bulk suggests an additional interfacial strain contribution from the pseudomorphic coherent growth under slight lattice‐mismatch conditions for the present Sc concentration. As support, geometric phase analysis (GPA) was conducted on stacked and aligned image data after non‐rigid registration, minimizing the effects of scan distortions and sample drift. The GPA strain data demonstrate the coherent in‐plane growth conditions near the interface as presented in Figure S2 (Supporting Information) and confirm larger out‐of‐plane strain located at the as‐grown interface. The observed change of the monolayer distance at the as‐grown interface is well correlated with the gradual change in Z‐contrast between pure Ga atomic columns and pure Al,Sc atomic columns, indicating an almost chemically sharp interface. A magnified view of the atomic structure across the as‐grown interface is displayed in Figure 4b which shows the repetition of (α*A* − β*B*)_*n*_ structural units for the hexagonal stacking of the M‐polar unit cell. In this notation, the Greek letters indicate the projected anion‐(N) atomic positions and the capital letters the cation‐(Ga, and Al,Sc) atomic positions.

Next, we examine the interface structures after ferroelectric switching. The presented HRSTEM micrographs show the interfaces after multiple polarization inversion cycles have been performed and a partial switch from the M‐polar state to the N‐polar state was performed last. After partial switching from the M‐polar to the N‐polar state, vertical domains with M‐polarity remain present as discussed before in context of Figure 3e. These areas allow to identify the atomic interface structure for the switched M‐polar domain displayed in Figure 4c,d and (Figure S3, Supporting Information). The HRSTEM micrographs demonstrate that structural coherence to the GaN template is fully recovered for the M‐polar domain interface after ferroelectric cycling. Analysis of the out‐of‐plane strain distribution showing an average of ‐4.1% close to the interface suggests that lattice‐relaxation takes place during polarization switching.

In contrast, the inversion to N‐polarity in the Al_0.92_Sc_0.08_N layer results in the formation of a horizontal H‐H‐type polarization discontinuity directly at the interface. The change in interface structure is evident from the image contrast observed along the interfacial polarization discontinuity in contrast to the as‐grown and switched M‐polar interfaces (compare Figure S3, Supporting Information). The depicted image of the interface is certainly affected by monolayer roughness, structural alterations and superposition effects along the film depth direction. However, undistorted areas which show an atomically sharp interface across a few to tens of nanometers were also identified. As an example, the HRSTEM micrographs presented in Figure 4e,f,g,h show two possible atomic configurations of this horizontal interface inversion domain boundary. For these interfaces, the calculated monolayer separation reveal a larger relative spacing Δ*d*(0002) ≈ +26% and ≈ +11.5% at the interface, respectively. In addition, the average monolayer separation Δ*d*(0002) between the GaN and the N‐polar Al_0.92_Sc_0.08_N monolayers is determined to be −4.6% and −3.8% for both interfaces, indicating strain relaxation at the interface by ferroelectric switching. Strain relaxation is rationalized by the interface reconstructions resulting in the loss of direct structural coherence across the interface as displayed in Figure 4f,h. Moreover, the HRSTEM images show interruptions of the hexagonal stacking sequence (α*A* − β*B*)_*n*_ observed for the as‐grown and switched M‐polar interfaces. Here, the stacking is changed to (*C*γ − *A*δ)_*n*_ in the Al_0.92_Sc_0.08_N monolayers with shifted nitrogen positions. Essentially, ferroelectric switching alters the atomic structure via a basal plane stacking fault which translates into a single cubic block with *A*‐*B*‐*C*‐type stacking across the interface. The observed huge increase of the monolayer distance at the interface shown in Figure 4f suggests the presence of an anion‐layer α′. This assumption is supported by the measured intensity profile within the gap showing evenly spaced peaks of intensity.

A second observed atomic configuration of a monoatomic interface inversion domain boundary is shown in Figure 4g,h. Again, a stacking fault along the basal planes is introduced changing the stacking scheme into *A‐B‐A'‐C‐B* across the interface, where the *A'* layer denotes the transition layer between M‐polar and N‐polar regions. The anion‐sub‐structure around the *A'* cation layer is tentatively proposed based on its similarity to the structural superposition motif frequently observed at the polar domain boundaries, i.e., with two projected anion positions on each side of the cation.^[^ ^33^, ^38^, ^41^ ^]^

In Figure S4 (Supporting Information), we propose a model of the atomic structure which is based on the observed interfacial H‐H boundary and the determined monolayer separations presented in Figure 4e,f. For simplicity, a chemically sharp separation of Ga and (Al,Sc) containing monolayers was assumed and impurities of silicon and oxygen were not considered (compare Figure S4, Supporting Information). The structural model suggests that the metal positions (layer C) in the first switched N‐polar monolayer are coordinated by six nearest neighbors of anions (‘N1’ belong to the anions α′ and ‘N2’ to γ, compare Figure S4b, Supporting Information) forming a distorted octahedron at the interface. These octahedra are connected to each other by common edges and by common corners to the neighboring tetrahedra. Octahedral coordination of the M(Al,Sc) cation is rarely found in other compounds but exists for instance in the high‐pressure phase AlP_6_O_3*x*_(NH)_3 − *x*_N_9_ and of course rock‐salt‐type Al_1‐x_Sc_x_N.^[^ ^42^, ^43^, ^44^ ^]^

The formation of planar inversion domain boundaries can be induced by impurity doping of AlN and ZnO thin films, in which the dopant (e.g., Mg, Sb) cations are present in octahedral coordination.^[^ ^38^, ^45^ ^]^ Moreover, it is known that a change of ionic contributions, e.g., via targeted oxygen incorporation into nitrides, can initiate polarization inversion during the growth of AlN‐based thin films.^[^ ^41^, ^46^, ^47^, ^48^ ^]^ However, despite a few initial reports, so far no open discussion on interfacial inversion domain boundaries in all‐nitride Al_0.92_Sc_0.08_N/GaN heterostructures, either accessed by the growth conditions itself or by ex situ ferroelectric switching is available up to date.^[^ ^49^, ^50^ ^]^

The chemical structures of the Al_0.92_Sc_0.08_N/GaN interfaces before and after switching were investigated using STEM‐EDS mapping and electron energy loss spectroscopy (EELS). The STEM‐EDS intensity profiles and the corresponding elemental maps (cf. Figure S5, Supporting Information) show the elevated signals of oxygen and silicon at the Al_0.92_Sc_0.08_N/GaN interface, with overall marginal intensity. Silicon is used as the n‐type dopant to increase the conductivity of the GaN template, whereas the oxygen signal could stem from imperfect substrate cleaning prior to sputter deposition and the affinity of silicon to attract oxygen. The total O‐K peak intensity at the interface is about twice as large as for the Al_0.92_Sc_0.08_N above the interface. Since the accumulation of oxygen species is known to be influential to the formation of polar inversion domain boundaries it can not be ruled out that it plays a role on the formation of the particular interfaces observed here.

**Figure 5:**
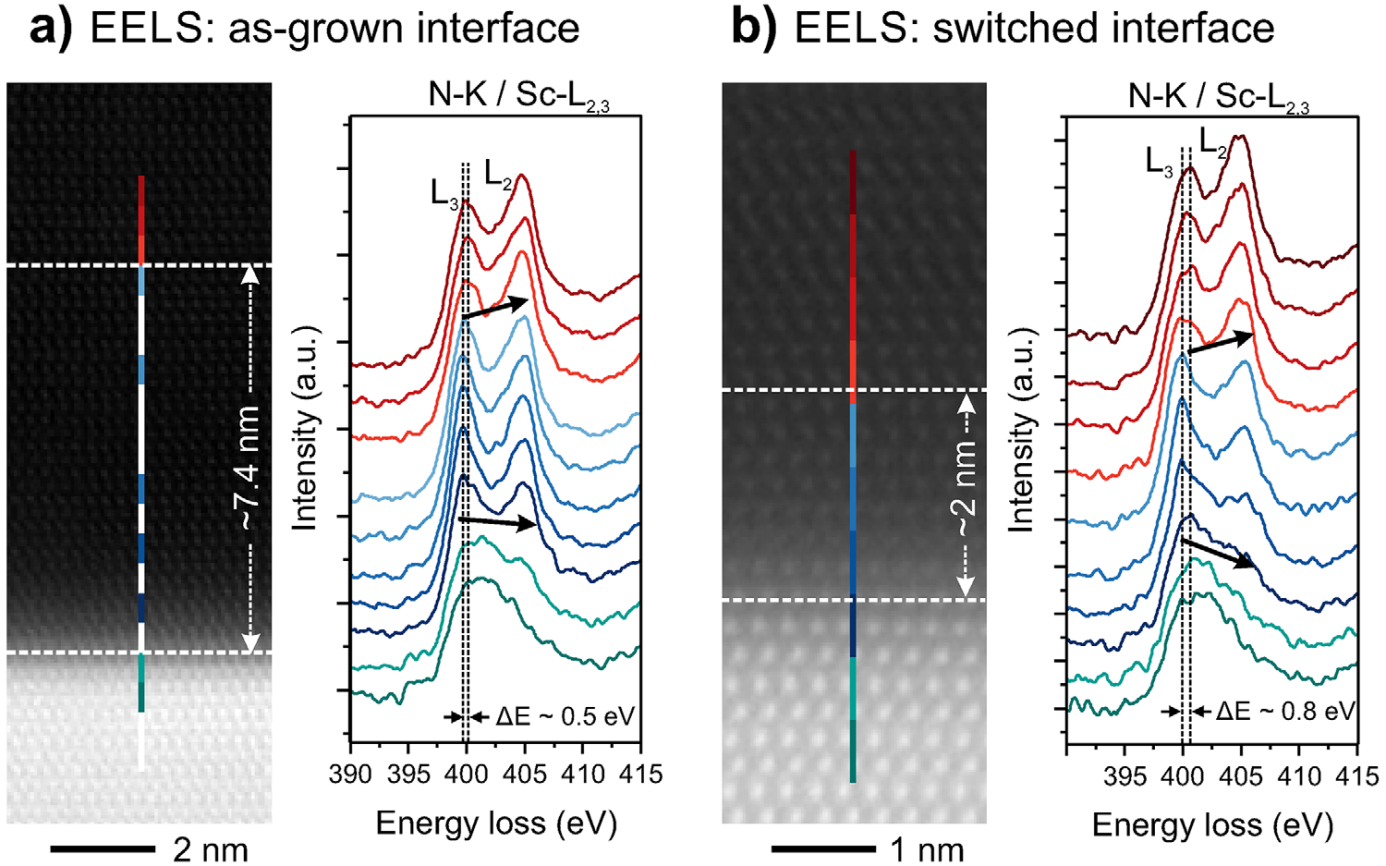
Spectroscopic analyses of the chemical structure at the Al_0.92_Sc_0.08_N/GaN interface. a) STEM‐EELS: Line‐profile measurement of the N‐K | Sc‐L_2, 3_ edge across the as‐grown interface. b) STEM‐EELS: Line‐profile measurement of the N‐K | Sc‐L_2,3_ edge across the switched N‐polar interface. Arrows indicate the change of intensity distribution between the two peaks.

Further, STEM‐EELS investigations of the electronic fine structure of the Sc‐L_2, 3_ edge across the as‐grown and switched interfaces was performed. EELS analysis of Al_1‐x_Sc_x_N naturally faces the difficulty of superimposed N‐K (402 eV) and Sc‐L_2, 3_ (402 and 407 eV) edges, which complicates data interpretation of fine structure variations. In addition, theoretically supported interpretations of the Sc‐L edge energy position and intensity distribution are not‐existent for alloys of Al_1‐x_Sc_x_N. The edge fine structure is assumed to be affected by the Sc‐concentration, strain distributions or the partial substitution of N by O or correlated vacancies.^[^ ^51^ ^]^ However, as visible in this study, the high‐intensity of the Sc‐L_2, 3_ peaks become already dominant over the N‐K edge intensity at low Sc concentrations of *x* = 0.08. For Al_0.92_Sc_0.08_N, the convoluted N‐K | Sc‐L_2, 3_ edge signal shows a characteristic double peak structure which is related to the defined transitions from the 2p^3/2^ and 2p^1/2^ levels into unoccupied 3d‐states (L_2, 3_) present for the transition metals, or so called white‐lines.^[^ ^52^ ^]^ In contrast, the N‐K edge for GaN only shows one broadened edge profile. For evaluation, the EEL spectra were binned over 3x1 pixels with a total length of 0.6 nm. The smoothed spectral data of the N‐K | Sc‐L_2, 3_ edges recorded across the as‐grown interface and the polarization discontinuity are presented in **Figure** **5**a,b, respectively.

At first, we noted that the edge energy is shifted of about ‐1 eV for Al_0.92_Sc_0.08_N with respect to AlN.^[^ ^53^ ^]^ In the case of the as‐grown interface, we observed that the characteristic white‐line edge profile of Sc‐L_2, 3_ is evolving from the interface to the bulk. Based on the spectral intensity distribution across the L_3_ and L_2_ peaks, two regions can be classified in the majority of our experiments. In the first region, i.e., the interface region, the L_3_ peak at lower energy loss exhibits a higher intensity than the L_2_ peak at higher energy loss; a situation thst is reversed in the second region, i.e., the bulk region, shows a higher intensity for the L_2_ peak. In addition, a small energy shift of about +0.5 eV of the L_3_ peak is observed with this transition. We noticed that the width of the interface region is in the range of ≈ 5–10 nm from the GaN interface in our experiments. Further, our EELS investigations across the reconstructed interfaces on M‐polar and N‐polar switched regions demonstrate that this extended interface region is reduced to a width of about 2 nm as presented in Figure 5b and Figure S6 (Supporting Information), or disappears completely. The locally recorded spectral data show a very similar distribution of peak intensities within the interface region and a clear transition to the bulk region which is accompanied with a slightly larger energy shift of the L_3_ peak of +0.8 eV. As mentioned above, at this point, we can only speculate on the origin of the observed intensity changes and energy shifts in the convoluted N‐K | Sc‐L_2, 3_ edge profile. Layers with Sc concentrations below the lattice matched concentrations are expected to suffer from strain relaxation due to the low critical thickness on GaN.^[^ ^54^ ^]^ Hence, in a reasonable assumption, we account for the observed energy shift as a result of the changed epitaxial strain distribution at the interface, leading to a change in the electronic structure by crystal‐field splitting.^[^ ^55^, ^56^ ^]^ However, confirmation of this claim as well as the intensity redistribution requires theoretical modeling.

To conclude, the discussed atomic interface structures and the implied pathway of interfacial inversion domain boundary formation require validation by theoretical studies and further observations. Unexpectedly, the observed interface reconstructions along the polarization discontinuities seem to be fully reversible when switching back from N‐polarity to M‐polarity. Furthermore, implications of the modified interface structures on the magnitude and screening effects of the polarization discontinuity and the sheet charge mobility and density in ferroelectric Al_1‐x_Sc_x_N/GaN‐based heterostructures requires further investigation.

### 2.4 Discussion: Switching Pathways in Al_1‐x_Sc_x_N/GaN Heterostructures Produced by MOCVD and Sputter Epitaxy

**Figure 6:**
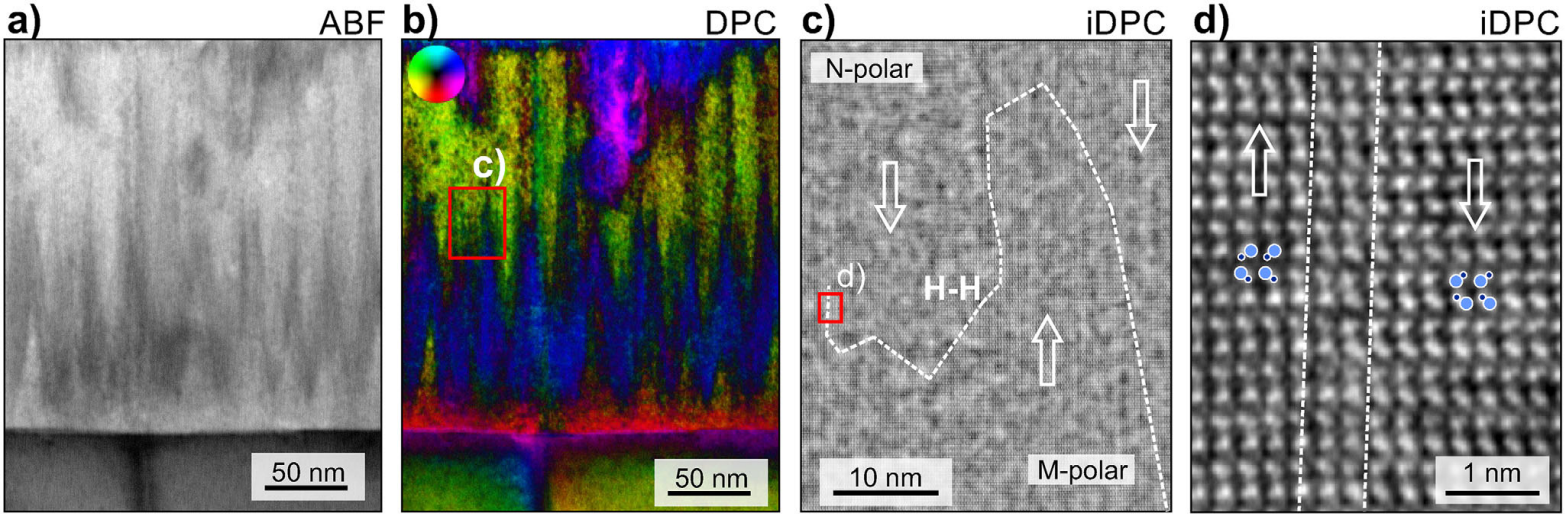
Ferroelectric domain structure in MOCVD‐grown Al_0.86_Sc_0.14_N thin film. a) ABF‐STEM image and the corresponding b) DPC‐STEM image highlighting the position of ferroelectric domain walls by the center‐of‐mass shift of the electron beam (blue). The blue color found at the upper part of the film presumably stems from crystalline defects. c) Large‐field‐of‐view iDPC imaging enables to identify the distribution of inclined domain walls between polar domains. The probed region is recorded at one of the tips of the blue spikes, as sketched in b), but is not identical to it. d) HRSTEM image of an inclined domain wall with limited thickness of 1–2 unit cells. The respective region is marked in (c).

To provide a direct comparison of the established domain patterns in sputtered and MOCVD‐grown layers, we revisit the latter system in **Figure** **6** for a short discussion. As outlined in previous works,^[^ ^33^, ^34^ ^]^ switching from M‐ to N‐polarity follows the vertical extension of wedge‐shaped domains to which propagation is stopped some tens of nanometers above the interface to GaN. There, the latticed‐matched MOCVD‐grown Al_0.86_Sc_0.14_N thin film has a highly coherent connection to the underlying M‐polar GaN. The ABF‐STEM image in Figure 6a and the DPC‐STEM image of Figure 6b show the zig‐zag pattern of the inclined domain walls formed in this system. The integrated (iDPC) signals may further improve image contrast and resolution that allows imaging of atomic structures with light elements and the identification of N‐ or M‐polarity in wurtzite‐type structures.^[^ ^57^ ^]^ By recording a large‐field‐of‐view iDPC micrograph with atomic resolution, a good correlation of the zig‐zag contrast with respect to the position of domain walls was achieved as sketched onto the micrograph displayed in Figure 6c. The dashed line indicates the position of the H‐H domain walls separating regions with N‐polarity (top) and M‐polarity (bottom). A magnified view onto the more vertical domain wall on the left side of the image is shown in Figure 6d. The displayed domain wall is approximately one nanometer thick.

**Figure 7:**
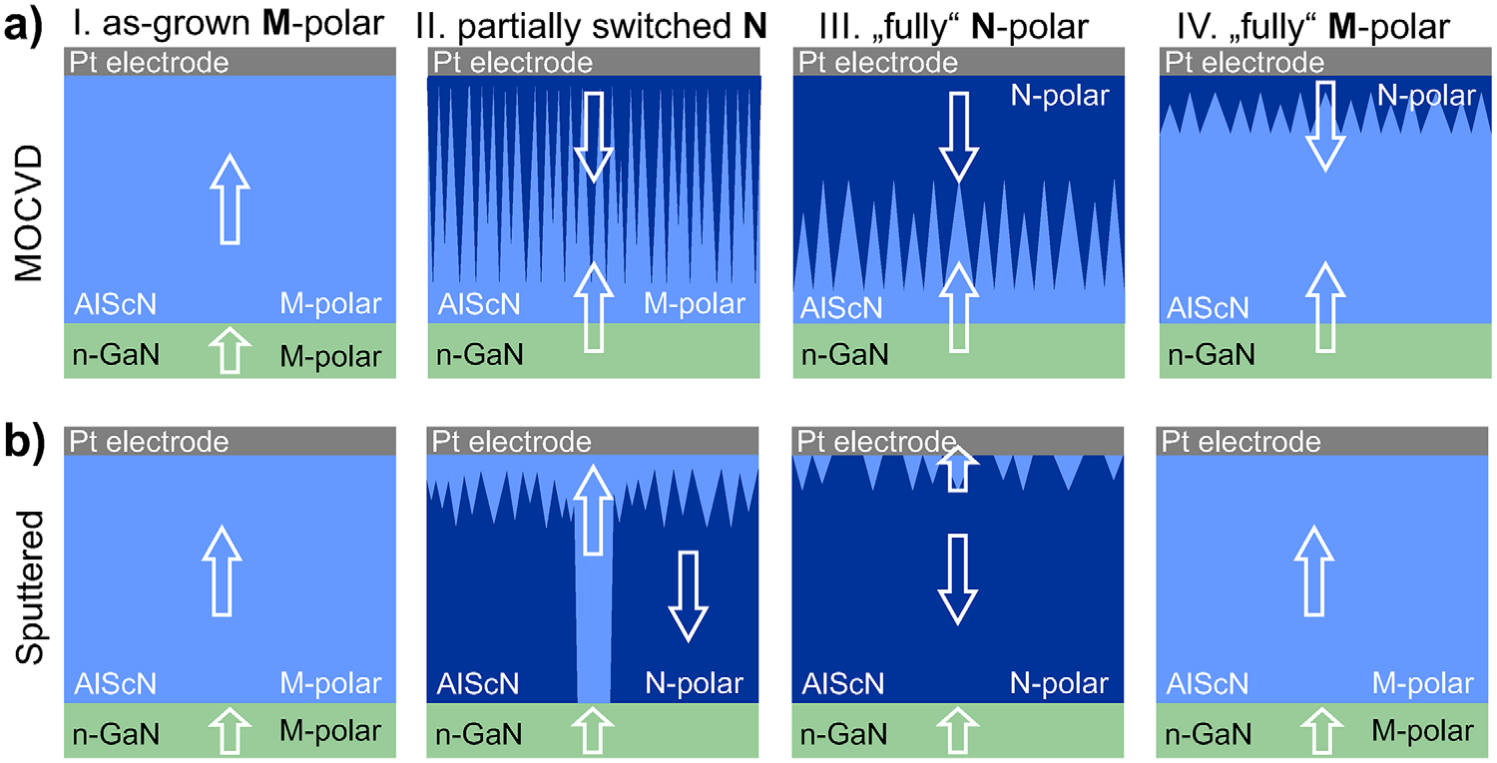
Sketches of the supposed switching pathways based on STEM observations: a) MOCVD‐grown Al_0.86_Sc_0.14_N and b) sputtered epitaxial Al_0.92_Sc_0.08_N thin films on GaN. The white arrows indicate the polarization vector of individual domains in the as‐grown partially and fully switched states. More details on the domain states for the MOCVD‐grown Al_0.86_Sc_0.14_N film are discussed in previous works.^[^ ^33^, ^34^ ^]^

The domain structure and the switching mechanism in MOCVD grown Al_0.86_Sc_0.14_N layers is presented in **Figure** **7**a and shows strong contrast to the domain structures observed in the sputtered Al_0.92_Sc_0.08_N layer (cf. Figure 7b). Whereas polarization inversion and domain propagation is observed to proceed from the top Pt interface in MOCVD‐grown layers, the ferroelectric M‐to‐N polarization inversion in sputtered epitaxial layers featuring local tensile strain is evidenced to be initiated at the GaN interface. As a result an atomically‐sharp horizontal H‐H polarization discontinuity is formed at the interface to the M‐polar GaN. N‐polarity extends through the bulk of the film and stops close to the Pt‐electrode interface, resulting in the stabilization of persistent fringed M‐polar inversion domain nuclei in both partially and fully switched films.^[^ ^39^ ^]^ We note that for sputtered films grown on metal layers and with a higher Sc content, polarization inversion was observed to commence from the top electrode interface.^[^ ^36^, ^58^ ^]^ Thinking of a subsequent N‐to‐M switching event, this observation could resonate with a schematically proposed domain structure model discussed for the ferroelectric ZnMgO.^[^ ^59^ ^]^ There, the application of a static electric field to the film surface by a scanning probe microscopy tip led to the proposed formation of surface‐near spike‐domains that break through individually to the substrate interface after passing a threshold bias field.

The crystal quality of MOCVD‐grown Al_0.86_Sc_0.14_N films and Al_0.92_Sc_0.08_N sputtered films differs significantly, as discussed in Figure 1. We hypothesize that for both cases the defect density and structural perfection determine the evolution of the observed domain patterns. At strongly coherent interfaces (MOCVD Al_0.86_Sc_0.14_N/GaN), the M‐to N‐polarization inversion is hindered while tensiley strained and defect‐rich interfaces (sputtered, Al_0.92_Sc_0.08_N ) can represent starting positions for domain nucleation.

Monoatomic step edges and non‐zero epitaxial strain at the GaN interface together with a more distorted and less coherent crystal structure in sputtered layers could promote polarization inversion at the GaN interface. In contrast, the nucleation of domains for MOCVD films starts close to the Pt interface where small variations of the Sc‐concentration were determined at vertically aligned defects.^[^ ^34^ ^]^

These defects could act as local atomic scale strain centers favoring the observed formation of inversion domains close to the top electrode interface. In conclusion, our direct examinations of ferroelectric domain structures support the observations that polarization switching in Al_1‐x_Sc_x_N is strongly interrelated with the nanostructural and chemical properties and its variations within the film.^[^ ^60^, ^61^ ^]^ In this respect, Yasuoka et al. demonstrated the impact of local lattice strain to initiate the ferroelectric switching by the introduction of additional interfaces and strong compositional gradients between individual layers within the ferroelectric.^[^ ^62^ ^]^ However, no attention has been paid to the impact of interface properties on ferroelectric switching pathways in epitaxial heterojunctions yet.

The presented understanding of domain structures in sputter deposited ferroelectric Al_1‐x_Sc_x_N thin films is highly relevant for the development of future ferroelectric non‐volatile memory devices which discriminate between individual polarization states.^[^ ^63^, ^64^ ^]^ Moreover, the revealed pathways of polarization inversion in epitaxial Al_1‐x_Sc_x_N/GaN heterostructures provide a starting point for the future development of functional ferroelectric all‐III‐N device such as FeHEMT.

## 3 Conclusion 

The development of wurtzite‐type ferroelectric requires fundamental understanding of the switching property interrelations with the crystallographic real structure. The multiscale structural properties of MOCVD and sputtered Al_1‐x_Sc_x_N/GaN heterostructures were investigated by XRD and aberration‐corrected STEM. By comparing the studied polarization domain structures after partial and fully ex situ switching events, significant differences in the switching pathways could be inferred. Engineering of the in‐plane lattice coherency in Al_1‐x_Sc_x_N/GaN heterojunctions via Sc‐concentration results in a tensiley strained ferroelectric interface layer. Hence, for the sputtered Al_0.92_Sc_0.08_N film the ferroelectric polarization inversion commences by reversible atomic reconstructions forming a planar interfacial H‐H domain wall at the GaN interface. In contrast, the assumably lattice‐matched MOCVD‐grown Al_0.86_Sc_0.14_N film having a highly coherent interfacial domain, shows the nucleation of inversion polarization domains with H‐H domain walls at the Pt electrode interface. Based on these findings, the significance of interface and defect engineering in wurtzite‐type ferroelectrics is evident, in the focus of scientific research on future ferroelectric non‐volatile memory devices, and novel ferroelectric III‐N heterostructures.

## 4 Experimental Details

### Thin Film Deposition

For the sputter‐deposited heterostructure commercially bought n‐type (Si) doped GaN(4 μ m)/Sapphire served as template for further film growth. The templates were cleaned with acetone and isopropanol using an ultrasonic bath, followed by rinsing with deionized (DI)‐water and drying with nitrogen gas. The 100 nm thin Al_0.92_Sc_0.08_N layer was deposited by pulsed‐DC co‐sputtering from Sc and Al targets at 450 °C under a N_2_ flow of 15 sccm, applying 140 W to the Sc target and 860 W to the Al target using an Oerlikon MSQ 200 multisource system. The 30 nm thin Pt layer was deposited after vacuum break via DC sputtering. Pt electrodes were structured via lithography and ion beam etching (IBE, Oxford Instruments Ionfab 300). Growth details on the ferroelectric Al_0.86_Sc_0.14_N film using MOCVD can be found elsewhere.^[^ ^33^ ^]^

### Ferroelectric Experiments

Electrical characterization and pre‐treatment prior to STEM investigations of individual capacitors were performed by applying a voltage signal with triangular waveform at 1.5 kHz using an aixACCT TF 2000 analyzer. The drive signal was applied to the top electrode. In this pre‐treatment, the sputtered Al_0.92_Sc_0.08_N layer was subjected to 20 complete polarization cycles, the MOCVD Al_0.86_Sc_0.14_N layer was subjected to 400 complete polarization cycles. Further details on the electrical pre‐treatment of the MOCVD‐grown Al_0.86_Sc_0.14_N thin film can be found elsewhere.^[^ ^33^, ^34^ ^]^

### Nanostructure Investigations

**XRD**: The X‐ray diffraction experiment (ω‐scan, RSM) was performed utilizing a Rigaku SmartLab (9 kW, Cu‐kα). The incident beam was monochromated with a Ge(220)2x monochromator and the diffracted beam was detected with a Hypix‐3000 pixel detector.

**TEM**: High‐resolution transmission electron microscopy (HRTEM) and high‐resolution scanning transmission electron microscopy (HRSTEM) imaging of the sputter deposited Al_0.92_Sc_0.08_N/n‐GaN heterostructure was conducted on a probe‐corrected transmission electron microscope (NEOARM / JEM‐ARM200F, JEOL) operated at the acceleration voltage of 200 kV. High‐angle annular dark‐field (HAADF, 80‐220 mrad) and annular bright‐field (ABF, 10‐20 mrad) STEM detectors were used for resolving the film polarity on the atomic level. Scan distortions and sample drift during image acquisition were minimized by fast serial recording of multi‐frame images followed by post‐processing image alignment. The rigid and non‐rigid image registration of serial image stacks was performed using the Smart Align ^[^ ^65^ ^]^ (HREM Research Inc.) plug‐in running on the DigitalMicrograph v.3.5.1 (DM) (GatanInc) software. Fourier‐filtering of non‐rigidly processed STEM micrographs was applied using a simple radiance difference filter (lite version of DM plug‐in HREM‐Filters Pro/Lite v.4.2.1, HREM Research Inc.) to remove high‐frequency noise from the post‐processed image. The monolayer separation across the interfaces was calculated by the Atomap toolbox using a 2‐D Gaussian peak fitting approach for finding atomic column positions.^[^ ^40^ ^]^ Electron energy loss spectroscopy (EELS) was performed after tuning the Continuum S spectrometer (Gatan Inc.) to an energy resolution of about 0.55 eV, using an entrance aperture of 5 mm and dispersion of 50 meV/channel. Spectral data was recorded using the dual‐EELS acquisition mode and serial acquisition along line profiles, thereby distribution the dose across several nanometers in width. Data analysis was done on the DM software. For plotting, the spectral data was smoothed using the Savitzky‐Golay approach.

Integrated differential phase contrast (iDPC) HRSTEM and EDS experiments were conducted on the double‐corrected transmission electron microscope (Themis Z, Thermo Fisher) operated at an acceleration voltage of 300 kV and equipped with the Super‐X EDS system. The electron beam deflection across domain walls was evaluated by calculating the center‐of‐mass (COM) shift of the scattered intensity via segmented detectors with collection angles between 6 –23 mrad. The convergence angle of the electron beam was 30 mrad. DPC mapping to detect the position of domain walls was performed using the electron beam with a convergence angle of 10 mrad and the collection angle range is 3 to 17 mrad.

## Conflict of Interest

The authors declare no conflict of interest.

## Author Contributions

N.W. led the conceptualization, methodology, formal analysis, investigation, original draft writing, and visualization, and contributed equally to project administration. G.S. supported the conceptualization and investigation and contributed equally to writing – review and editing. R.I. supported the investigation and contributed to writing –review and editing. Z.D. supported the investigation and contributed equally to writing – review and editing. C.K. provided supporting resources and contributed equally to writing – review and editing. S.F. provided supporting resources, contributed equally to writing – review and editing and project administration, and shared responsibility for funding acquisition. L.K. also provided supporting resources, contributed equally to writing – review and editing and project administration, and shared responsibility for funding acquisition.

## Supporting information

Supporting Information

## Data Availability

The data that support the findings of this study are available from the corresponding author upon reasonable request.
