# Supplementary material for: Interfacial Polarization Switching in Al0.92Sc0.08N/GaN Heterostructures Grown by Sputter Epitaxy
Source: Adv Sci (Weinh). 2025 Jun 6;12(30):e03827. doi: 10.1002/advs.202503827 (PMC12376545; doi:10.1002/advs.202503827)
Supplement: Supplementary file 1 — Supporting Information [file ADVS-12-e03827-s001.pdf]

ADVANCED SCIENCE
Open Access

Supporting Information

for *Adv. Sci.*, DOI 10.1002/advs.202503827

Interfacial Polarization Switching in $Al_{0.92}Sc_{0.08}N$/GaN Heterostructures Grown by Sputter Epitaxy

Niklas Wolff, Georg Schönweger, Md. Redwanul Islam, Ziming Ding, Christian Kübel, Simon Fichtner and Lorenz Kienle*

Supporting Information

Interfacial Polarization Switching in $Al_{0.92}Sc_{0.08}N$/GaN Heterostructures Grown by Sputter Epitaxy

Niklas Wolff[*1,2], Georg Schönweger[1,3], Redwanul Islam[1], Ziming Ding[4,5], Christian Kübel[4,5], Simon Fichtner[1,2,3], Lorenz Kienle[1,2]

[1] Department of Material Science, Kiel University, Kaiserstrasse 2, D-24143 Kiel, Germany

[2] Kiel Nano, Surface and Interface Science (KiNSIS), Kiel University, Christian-Albrechts-Platz 4, D-24118 Kiel, Germany

[3] Fraunhofer Institute for Silicon Technology (ISIT), Fraunhoferstr. 1, D-25524 Itzehoe, Germany

[4] Advanced Electron Microscopy in Materials Research, Institute of Nanotechnology (INT), Karlsruhe Institute of Technology (KIT), D-76344 Eggenstein-Leopoldshafen, Germany

[5] Karlsruhe Nano Micro Facility (KNMFi), Karlsruhe Institute of Technology (KIT), D-76344 Eggenstein-Leopoldshafen, Germany

Correspondence

* Niklas Wolff,

Email: niwo@tf.uni-kiel.de

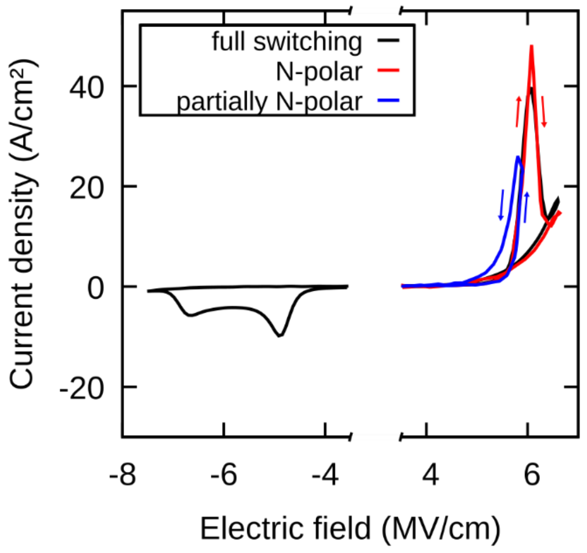

Figure S1: Electrical pretreatment and ferroelectric response of the Pt/$Al_{0.89}Sc_{0.11}N$/n-GaN-based capacitor prior to the investigations performed by STEM. The N-polar state was induced by inverting the polarization fully for twenty times (black) followed by the application of an unipolar voltage signal with 73 V amplitude (red). Similarly, the partially N-polar state was induced by pre-cycling a pristine capacitor for twenty times followed by the application of an unipolar voltage signal with 65 V amplitude to switch from the full M-polar state into partial N-polarity (blue). Note the counterclockwise current peak indicated by the blue arrows, which can be explained by the nucleation of inversion domains at high electric fields which still grow when sweeping the field back to lower values.[1] The broadened switching regime at negative fields (black) featuring two switching peaks is likely related to depletion of the n-GaN and the occurrence of low and high energy nucleation centers for polarization inversion e.g. due to an inhomogeneous strain/stress distribution.[2]

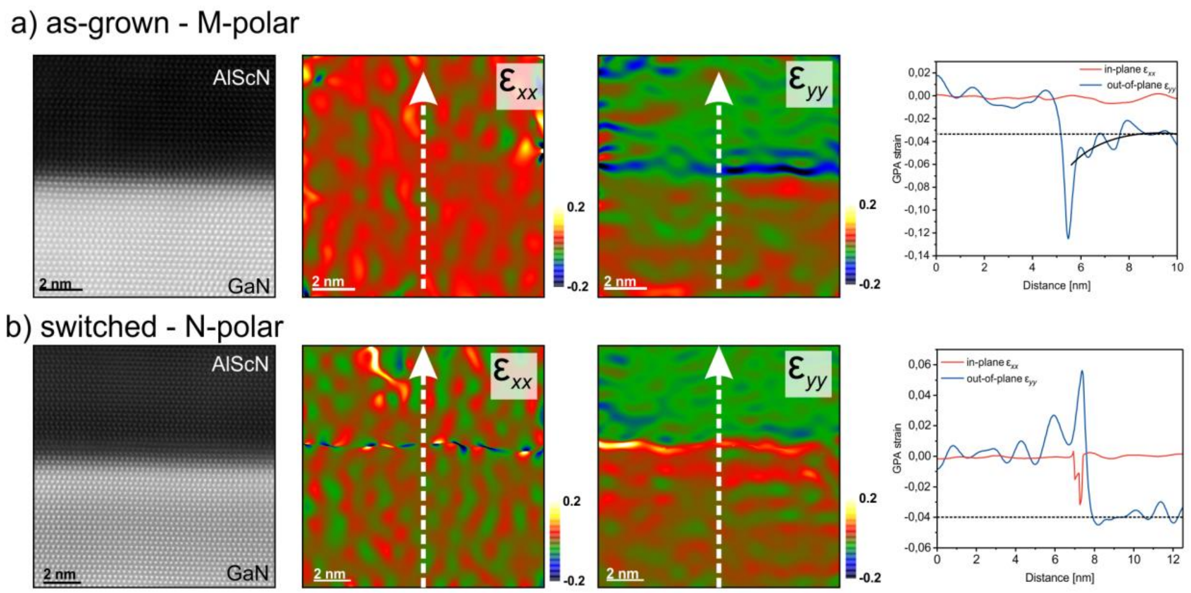

Figure S2: Strain analysis of the as-grown and pristine interfaces using the geometric phase analysis method. The calculated in-plane strain and out-of-plane distributions remarkably differ after ferroelectric switching. For the as-grown interface, a pseudomorphically strained interface is apparent with no in-plane strain relaxation at the interface or the layer. In film growth direction compressive out-of-plane strain relaxation is observed at the interface, congruent to the observations discussed in context of Figure 4a. For the switched interface, the introduced polarization discontinuity becomes apparent in the in-plane strain map showing compressive strain at the interface and rapid strain relaxation. Moreover, in the out-of-plane strain map, the accumulation of local tensile strain is evident at the polarization discontinuity, followed by rapid relaxation to -4 %.

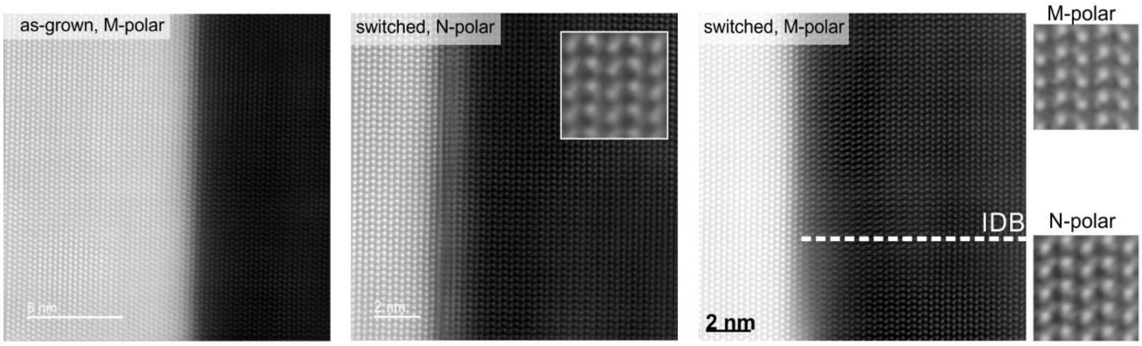

Figure S3: HRSTEM images of as-grown and switched AlScN/GaN interfaces.

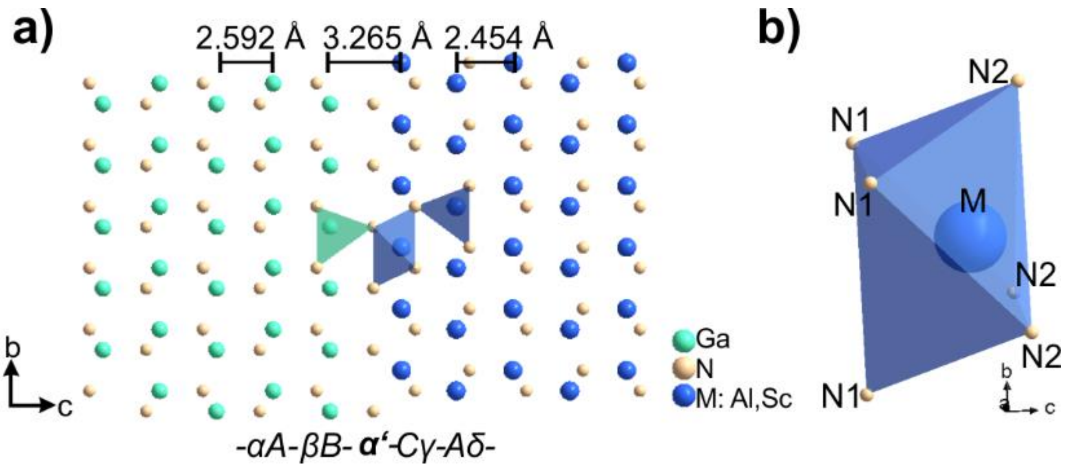

Figure S4: Structure model of the interfacial inversion domain boundary discovered in d,e). Distorted MN_6 (M: Al,Sc) octahedra are present at the interface, as shown in b). 'N1' labeled atomic positions correspond to the anion layer α.

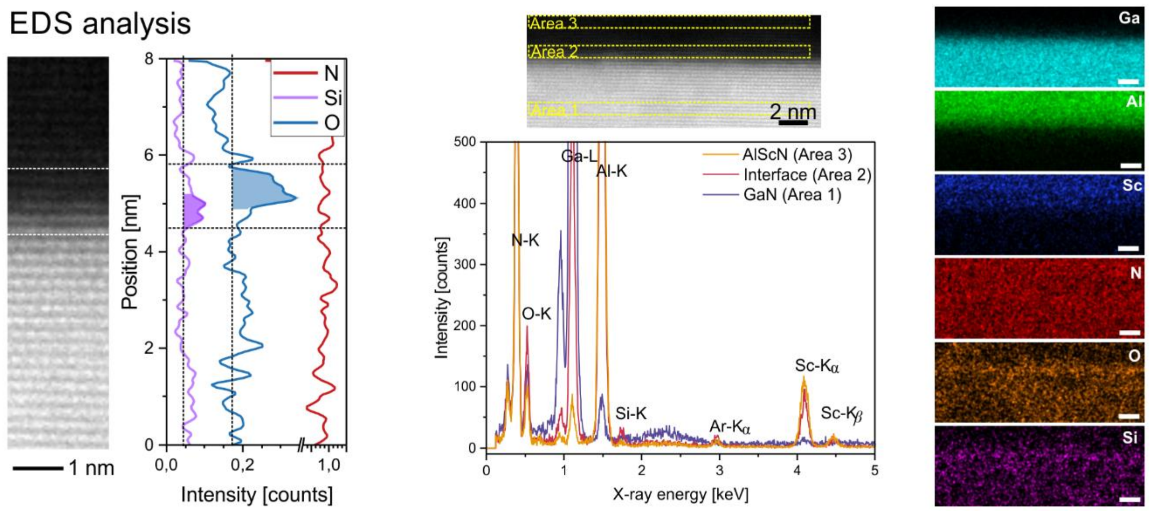

Figure S5: STEM-EDS analysis of the switched AlScN(N-polar) / GaN(M-polar) interface. The analysis demonstrates minor oxygen contamination at the interface.

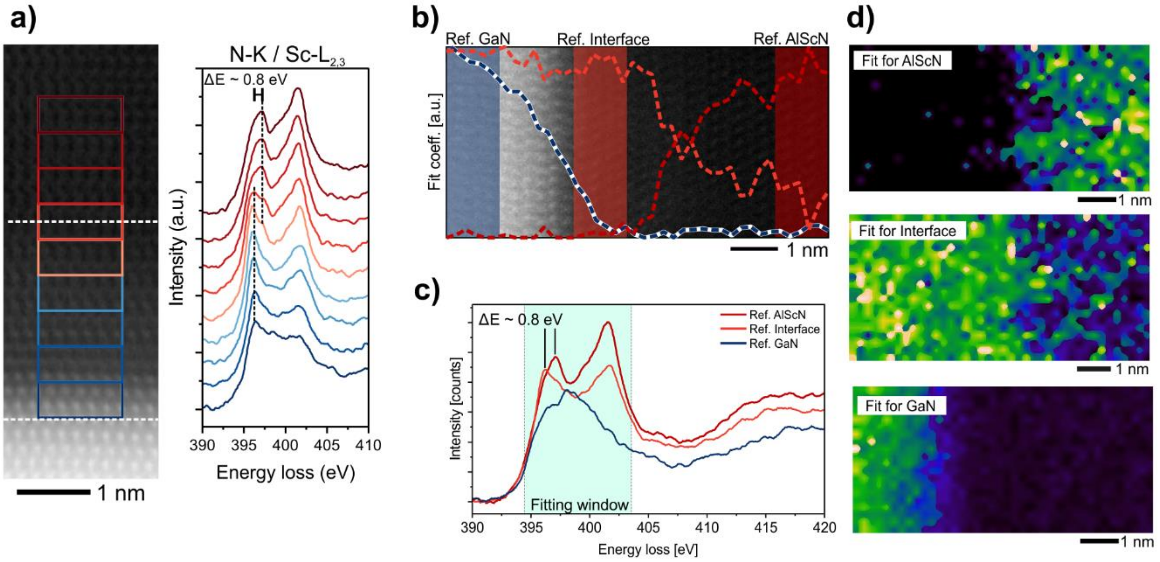

Figure S6: STEM-EELS measurement across the polarization discontinuity at the switched N-polar AlScN/GaN interface. a) Line profile measurements with summed data within each frame. A clear transition of the edge intensity profile accompanied with a 0.8 eV energy shift is observed approaching the bulk. b) EELS spectrum imaging across the interface. Three reference edge profiles were identified from the GaN, the interface and the bulk (compare c) and fitted to their local distribution using a multiple linear least-square fit routine (compare d). The profiles in b) show the goodness of the fit across the interface.

References:

[1] Gremmel M, Fichtner S. The interplay between imprint, wake-up, and domains in ferroelectric Al0.70Sc0.30N. Journal of Applied Physics. 2024;135(20):204101. doi: 10.1063/5.0197111.

[2] Schönweger G, Petraru A, Islam MR, et al. From Fully Strained to Relaxed: Epitaxial Ferroelectric Al1–xScxN for III-N Technology. Advanced Functional Materials. 2022;32(21):2109632. doi: https://doi.org/10.1002/adfm.202109632
